# Everybody's Page

**Published:** 1892-04-09

**Authors:** 


### Everybody's Page.

An American physician recommends those who suffer from asthma to drink strong coffee at their meals, or whenever they require liquid.

What a delightful sensation it must be to find money a superfluous encumbrance. There is one fortunate individual in the world who experiences the untold charms of this sensation, and this is M. Jacques, the fasting man, who, in reply to Judge Bayley's query as to what he was living on, stated that he is a fasting man, and lives on nothing. Happy and enviable man!

The mosquito comes in for a good deal of vituperation, but it appears not always justly. The male insect is asserted to be a gentleman in every sense of the word, as modest and inoffensive as he is retiring, whereas his consort should be blamed for her obtrusive and objectional habit of biting and stinging. A difference between the sexes which some people aver is not peculiar to the mosquito.

The *Medical Record* believes, that the days of the anti-vivisectionists are numbered, though they will probably die hard. Time alone can show whether the view is correct, that the public will side with the vivisectionists as soon as they are able to present in demonstrable form the knowledge they claim to be gleaning of the causes of the various contagious diseases which afflict the world.

A revised bill of fare has been issued for prisoners. A breakfast of bread and cocoa, dinner of bread and beef or mutton, and supper of bread and a pint of porridge ought to tempt lots of starving people to do something towards earning a spare meal at the expense of the State. But this bill of fare is of the nature of a snare and a delusion, as it is only a preliminary to the old friend "skilly," which still remains the ordinary prison diet.

For us who are the victims of every kind of weather, but more particularly of one sort, it is hard to realise what a pleasure life must be in a country where during the hottest day in the year the thermometer does not rise above 94 deg., and where there is not sufficient frost during the winter to kill potatoes left in the ground where they have grown. We are always grumbling at our so-called climate, but after a lengthened absence we are delighted to expose ourselves once more to its miseries.

Following up the discovery which was made many years ago, that the blood of rats, who are not subject to anthrax, possess properties which give immunity from the disease to other animals if the blood is introduced into their system, two German doctors are reported to believe themselves in a position to combat successfully the ravages of diphtheria. Their hope is based, however, on a hypothesis which has yet to be proved, that there is a practical ideality of organism in men and animals.

Mr. Justice Vaughan Williams in congratulating the grand jury at Chester Assizes on the remarkable absence of crime due to drink, and expressing a hope for a succession of such assizes which might lead to a reasonable anticipation of permanent improvement in the morals of the people, is reported to have said there is only one crime which can be attributed to drink—which crime is not stated. Many people think that the crimes which can be attributable to drink exceed in number and magnitude those which cannot be so attributed.

According to a German writer, the proper temperatures at which water, milk, red wine, champagne, and coffee should be drunk are respectively 54, 64, 66, 46, and 73 to 79 degrees.

A precise definition has been given to the term "total helplessness" in the United States. Any person is considered to be in this negative state of existence who has lost an arm or a leg so close to the shoulder or the hip that an artificial limb cannot be worn.

The bitterest detractors of the Salvation Army and its methods cannot accuse its organisers of want of ingenuity or originality. One of the latest notices issued by them reads as follows: "Safety matches are now made by the social wing without sulphur or phosphorus, which will flame without striking. What do we mean! Just this: That if you are unmarried and do not know where to choose a partner, you can communicate with Colonel Barker, Matrimonial Bureau, 101, Queen Victoria Street, E.C., and he will most probably supply you with what you want—somebody loveable and good." Colonel Barker ought to be a busy man.

The difficulties in the way of abating the smoke and fog nuisance in the metropolis seem hopelessly insuperable. Lord Midleton has received scant encouragement in his efforts, and it is now found impossible to obtain a Committee of the House of Lords which can attack the subject. The Duke of Westminster is asserted to plead as a reason why he cannot assist in investigating the matter that he is unable in ordinary seasons to breathe the air of London for any length of time. This ability to sympathise with the unfortunate Londoner is an additional qualification for the post of Chairman of such a Committee which it is regrettable the Duke cannot put to a practical test.

Much dissatifaction has been expressed in "sporting circles" at the action taken by the managers by some of our free libraries in "blacking" all sporting information from the papers entering the reading room. Seeing that free libraries are not intended for the use of any one section of society to the exclusion of others the sporting gentlemen have no cause for this dissatisfaction. As they can presumably afford the luxury of racing they can also afford to purchase their especial literature without interfering with the comfort of their neighbours, who will gladly dispense with the honour of their company in libraries. If other managers will follow the examples set them in this direction the libraries may yet become the useful educators which it was reasonably hoped they would prove.

An American writer maintains that æsthetic dress has become a strong factor in children's discomfort and disease. The good which has been done in the direction of substituting soft fabrics, light and suitable, as drapery for the heavier and more cumbersome textiles which, as he graphically puts it, made women blots on the scene instead of ornaments, has been counterbalanced by the harm inflicted, through the æsthetic craze, on children. The large hats and long gowns and coats which some people put their children into are marked down for his especial animadversion. In a wind a tottering child has to devote its tiny energies to its hat, with the result that its totter, aggravated by a long frock twining round its legs, is made still more unsteady, and becomes its natural gait. As a remedy for this unsatisfactory state of affairs, it is suggested that the fathers of such children should be put in long clothes and broad hats for a month. This month's imprisonment would of course entail with it such hard labour as would no doubt impel the parent to change his children's manner of dress.

